# A Review of Mechanical Properties and Rockburst Investigation of Transversely Isotropic Rocks by Experimental Technique

**DOI:** 10.3390/ma16083183

**Published:** 2023-04-18

**Authors:** Xuefeng Si, Song Luo, Yong Luo

**Affiliations:** 1 School of Resources Environment and Safety Engineering, University of South China, Hengyang 421001, China; xuefsi@usc.edu.cn (X.S.); luoyongcsu@163.com (Y.L.); 2 School of Resources and Safety Engineering, Central South University, Changsha 410083, China

**Keywords:** rock mechanics, transversely isotropic rocks, mechanical properties, failure mode transformation, rockburst

## Abstract

Under complex geostress caused by long-term geological evolution, approximately parallel bedding structures are normally created in rocks due to sedimentation or metamorphism. This type of rock is known as transversely isotropic rock (TIR). Due to the existence of bedding planes, the mechanical properties of TIR are quite different from those of relatively homogeneous rocks. The purpose of this review is to discuss the research progress into the mechanical properties and failure characteristics of TIR and to explore the influence of the bedding structure on the rockburst characteristics of the surrounding rocks. First, the P-wave velocity characteristics of the TIR is summarized, followed by the mechanical properties (e.g., the uniaxial compressive strength, the triaxial compressive strength, and tensile strength) and the related failure characteristics of the TIR. The strength criteria of the TIR under triaxial compression are also summarized in this section. Second, the research progress of the rockburst tests on the TIR is reviewed. Finally, six prospects for the study of the transversely isotropic rock are presented: (1) measuring the Brazilian tensile strength of the TIR; (2) establishing the strength criteria for the TIR; (3) revealing the influence mechanism of the mineral particles between the bedding planes on rock failure from the microscopic point of view; (4) investigating the mechanical properties of the TIR in complex environments; (5) experimentally investigating the rockburst of the TIR under the stress path of “the three-dimensional high stress + internal unloading + dynamic disturbance”; and (6) studying the influence of the bedding angle, thickness, and number on the rockburst proneness of the TIR. Finally, some conclusions are summarized.

## 1. Introduction

Under complex geostress caused by long-term geological evolution, approximately parallel bedding structures are developed in a rock mass due to sedimentation or metamorphism. This type of rock is known as the transversely isotropic rock (TIR). The TIR, such as phyllite, shale, schist, gneiss, and slate, is often encountered in underground engineering in central and western China [1]. Due to the presence of bedding planes, the mechanical properties of TIR are quite different from those of relatively homogeneous rocks. The failure behavior of TIR is closely related to the related mechanical properties of the rock and is influenced by the bedding angle [2,3,4,5], confining pressure [6,7,8,9], water content [10,11,12], and temperature [13,14,15]. In addition, the failure mechanism of TIR was also revealed [16,17,18]. Under uniaxial compression, the uniaxial compressive strength of TIR shows a U-shaped trend, first decreasing and then increasing with an increasing bedding angle [2,4]. The dynamic strength of the slate exhibits a higher anisotropy under dynamic loading, and the strength anisotropy index first decreases and then increases as the loading level increases [5]. Under triaxial compression, when the confining pressure is constant, the triaxial compressive strength of TIR shows a U-shaped variation trend with an increasing bedding angle. As the confining pressure increases, the triaxial compressive strengths at different bedding angles increase [7,8,9]. Tien et al. [17] and Wang et al. [18] found that the sliding failure along the layer depends on the direction of the bedding plane, the confining pressure, and the strength ratio of layered rock to intact rock. Their results indicated that the material and cementation degree of the bedding plane had an important influence on the mechanical properties of TIR. Additionally, TIR in water-rich formations can be affected by the water content [11,12]. With increasing water content, the mechanical properties and brittleness of the phyllite are weakened and the macroscopic failure angle increases, resulting in a decrease in tensile failure and an increase in shear failure [10]. Thermal treatment also has an important influence on the mechanical properties and failure modes of TIR [13,14,15]. For example, within the temperature range of 0 to 200 °C, the peak stress increased with the temperature when *θ* = 0°, 45°, and 90°. However, the peak stress decreased with an increasing temperature when *θ* = 60° [14]. Therefore, the above-mentioned factors have a significant influence on the transversely isotropic rock mass in underground engineering [19,20,21]. As the mining depth increases, rockburst can occur in a deep, transversely isotropic rock mass. For example, rockburst has occurred in a 3.5 m diameter TBM tunnel at the Kobbelv hydropower scheme in northern Norway [20], the Erlang Mountain tunnel [22], and the Tengyue tunnel in the southern segment of the Gaoligong Mountain [23,24]. A rockburst disaster poses a serious threat to the safety of construction personnel and equipment [25,26,27,28,29]. Many scholars have conducted investigations of the rockburst and brittle failure of tunnels and caverns on TIR or rock-like materials [30,31]. Sagong et al. [30] investigated the joint slip characteristics of a circular hole. Fortsakis et al. [31] described the engineering geological behavior of TIR in tunnels and analyzed the effect of the bedding structure on tunnel convergence. Si et al. [32] conducted the true triaxial test on the cubic phyllite samples with a circular hole under different bedding angles to investigate the influence of the bedding angle on the failure characteristics of the surrounding rock of the hole. In the above studies, only the conventional compression test and the simple simulation test were carried out. However, in deep underground engineering, a rock mass is in a complex stress environment, and the stress path can be described as “three-dimensional high stress + excavation unloading + stress adjustment (dynamic disturbance)” [33,34]. Research on deep underground engineering should be consistent with the stress path of the underground rock mass and the environment of the rock mass as much as possible, so that the research results can be well applied to engineering practice.

In order to discuss the development of the mechanical properties and failure characteristics of TIR, and to explore the influence of bedding on the rockburst of the surrounding rock, the research methodology in this paper includes three stages. The first is to collect experimental research literature on TIR using the Web of Science Core Collection database and China National Knowledge Infrastructure Academic Platform. The second is to classify and organize the collected literature (including the uniaxial compression, uniaxial tension, triaxial compression, rockburst simulation, and strength criteria). The third one is to analyze the shortcomings of the existing research and to propose research perspectives. This review consists of five sections: After the “Introduction”, in Section 2, the characteristics of the P-wave velocity of the TIR are summarized; then, the mechanical properties and failure characteristics under uniaxial compression are presented, and the tensile strength of the TIR is summarized; and finally, the mechanical properties and strength criteria of the TIR under triaxial compression are presented. In Section 3, the progress of the rockburst tests on the TIR is reviewed. In Section 4, six perspectives on the study of transversely isotropic rocks are proposed, which provide directions for the testing of the mechanical properties of TIR formations and the study of rockburst simulation experiments. Section 5 presents a brief summary and some prospective investigations.

## 2. Influence of Bedding Angle on Mechanical Properties and Failure Behavior

**Figure 1:**
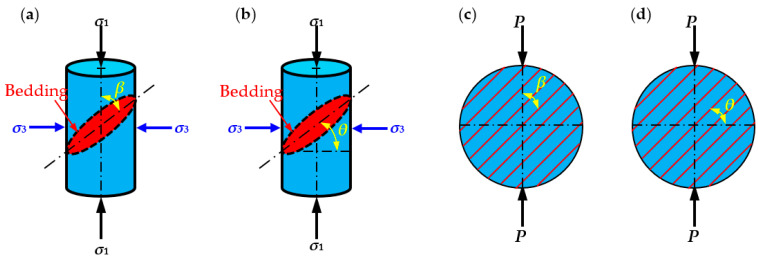
Schematic diagram of two definitions of the bedding angle: (**a**,**b**) definitions of the bending angle in uniaxial compression; and (**c**,**d**) definitions of the bending angle in tensile test.

Compared with intact rock, TIR has a bedding plane structure, which is the fundamental reason for the variation in the mechanical properties and failure mode of TIR. Therefore, the influence of the bedding angle has been of great interest to researchers. The mechanical properties of TIR mainly include the uniaxial compressive strength, triaxial compressive strength, and tensile strength. The failure characteristics of rock mainly refer to the failure mode and severity of the rock under different test conditions. In many studies, there are two definitions of the bedding angle: one is *β* between the bedding plane and the direction of the maximum principal stress *σ*_1_ (Figure 1a) or the tensile load *P* (Figure 1c), and the other is *θ* between the bedding plane and the direction of the minimum principal stress *σ*_3_ (Figure 1b) or the horizontal direction (Figure 1d). Figure 1 shows that under the same bedding conditions, the bedding angles under the two definitions have a certain correlation, i.e., *β* + *θ*=π/2.

### 2.1. P-Wave Velocity Characteristics of TIR

The effect of the bedding angle on the physical properties of TIR is mainly manifested in the influence on the wave velocity. Many studies show that the P-wave velocity of samples gradually decreases as the bedding angle increases [35,36,37,38]. The main reason is that the reflection and refraction of ultrasonic waves at the bedding plane dissipate some of the wave energy, resulting in a weakening of the wave’s ability to propagate [1,39]. However, some researchers have come to different conclusions [14,40]. Li et al. [40] found that the P-wave velocity is lower when *β* = 45°, while the P-wave velocities are higher at 0° and 90°. Guo et al. [14] found that the P-wave velocity increases with the bedding angle. The filling material in the bedding plane is of different properties and its development and extension are directional, which causes the coal and rock to have anisotropic acoustic characteristics [40].

### 2.2. Mechanical Properties and Failure Characteristics under Uniaxial Compression

**Figure 2:**
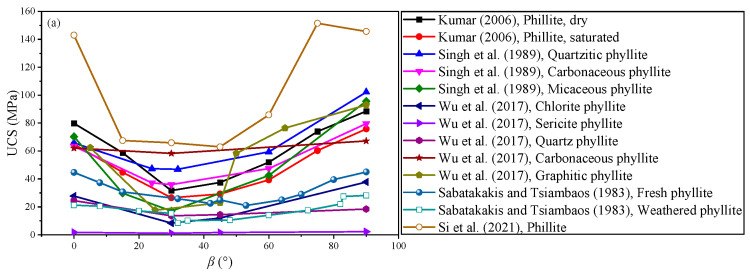

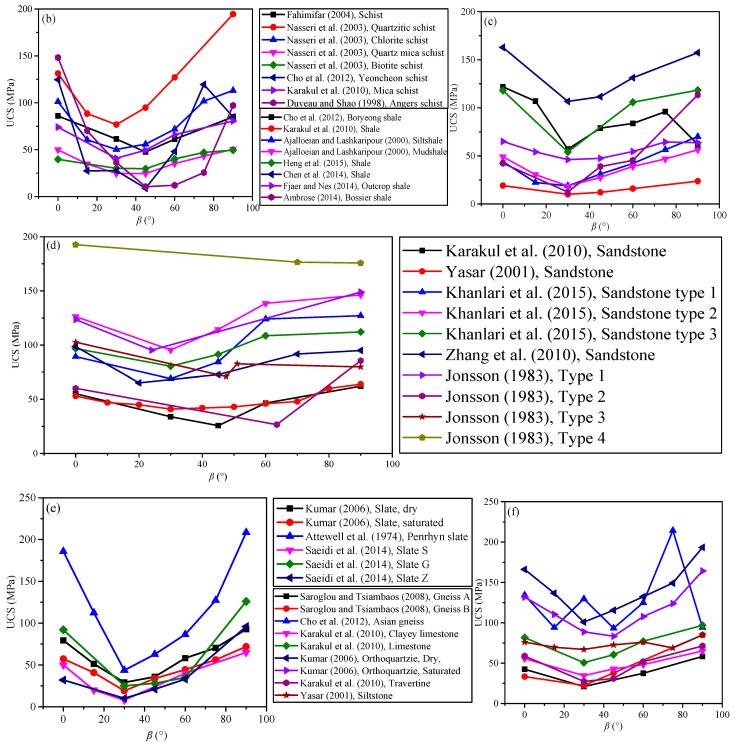
The relationship between the UCS and bedding angle of TIR: (**a**) phyllite [1,46,47,48,49], (**b**) schist [50,51,52,53,54], (**c**) shale [35,52,53,55,56,57,58], (**d**) sandstone [53,59,60,61,62], (**e**) slate [46,63,64], (**f**) gneiss [52,65], limestone [53], orthoquartzie [46], travertine [49], and siltstone [59].

**Figure 3:**
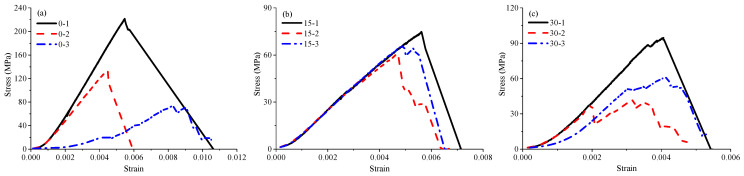

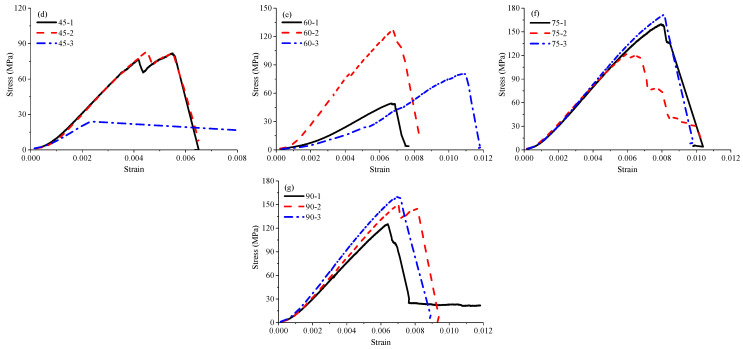
Stress–strain curves of phyllite specimens: (**a**) *β* = 0°, (**b**) *β* = 15°, (**c**) *β* = 30°, (**d**) *β* = 45°, (**e**) *β* = 60°, (**f**) *β* = 75°, and (**g**) *β* = 90° [1].

To investigate the relationship between the UCS and the bedding angle, the statistically obtained data from the uniaxial compression tests of transversely isotropic rocks (phyllite, schist, shale, sandstone, slate, gneiss, limestone, orthoquartzie, travertine, and siltstone) are plotted in Figure 2. The UCS first decreases and then increases with the bedding angle. In fact, the strength variation in typical TIR can be classified into three groups, namely the ‘‘U’’ type, ‘‘undulatory’’ type, and ‘‘shoulder’’ type [41,42,43]. Due to the influence of the bedding surface, the pre-peak or post-peak stress–strain curves of TIR in the uniaxial compression test can produce a small local stress drop (see Figure 3) [1,39,40]. The stress–strain curves can be divided into three types: rapid drop, sawtooth, and step drop [1]. As shown in Figure 3, the stress–strain curves of specimens 0-1, 0-2, 15-1, 30-1, 45-3, 60-2, 60-3, 75-3, and 90-3 are of the rapid-drop type; the stress–strain curves of specimens 0-3, 15-3, 30-2, 45-1, 45-2, and 90-2 are of the sawtooth type; and those of specimens 0-3, 15-2, 30-2, 30-3, 60-1, 75-1, 75-2, and 90-1 are of the step-drop type. Due to the presence of weak bedding planes in layered rocks, the specimen will undergo slip failure along the weak bedding planes, resulting in stress–strain curves with sawtooth or step-drop characteristics. For the same bedding angle, the peak stress of the rapid-drop stress–strain curve was the highest, followed by that of the sawtooth-type curve, and that of the step-drop stress–strain curve was the lowest.

**Figure 4:**
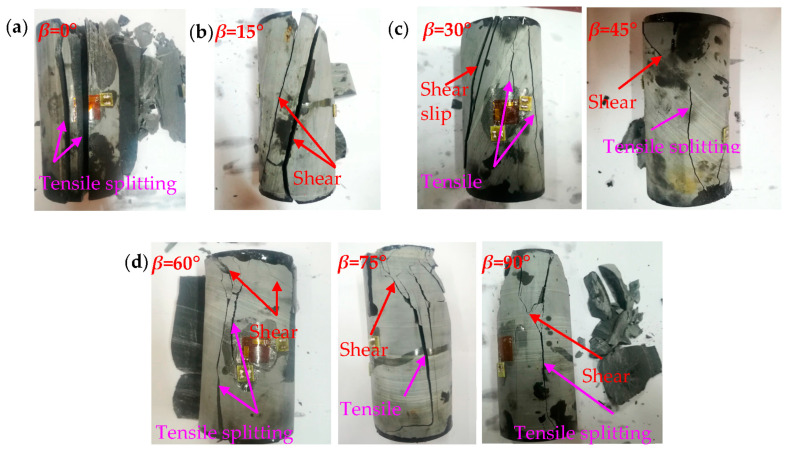
Failure modes of phyllite specimens with different bedding angles [1]: (**a**) 0°, (**b**) 15°, (**c**) 0° and 45°, and (**d**) 60°, 75° and 90°.

The strength anisotropy of TIR is inseparable from the failure mode [44]. The failure modes of transversely isotropic rocks under different bedding angles can be divided into four types: (a) tensile splitting failure along the bedding plane, (b) shear slip failure along the bedding plane, (c) a mixed failure mode of the tensile splitting failure across the bedding plane and shear slip failure along the bedding plane, and (d) tensile splitting failure across the bedding plane, as shown in Figure 4 [1,45]. Due to the presence of bedding planes, different failure modes are produced under different bedding angles, which in turn leads to strength anisotropy in TIR. The fundamental reason for the change in the UCS of TIR under different bedding angles is the transformation of the failure mode. There are many factors that affect the compressive strength of TIR. From the perspective of the rock itself, there are three important factors: the mineral composition, the cementation strength between mineral particles, and the bedding angle. For TIR, scholars mainly pay attention to the influence of the bedding angle. Considering the influences of the rock mineral composition and cementation strength between mineral particles, the compressive strength of the same type of TIR can also be analyzed, as shown in Figure 2. The results show that the difference in the compressive strength of similar rocks with the same bedding angle is mainly due to the various cementation strengths between the mineral particles. More experimental studies are needed to further verify this.

### 2.3. Tensile Strength of TIR

**Figure 5:**
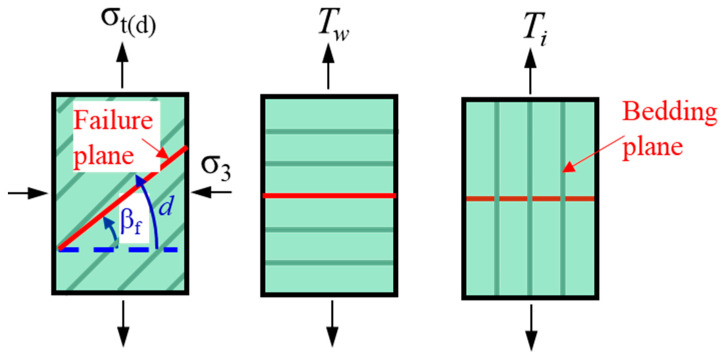
The tensile strength of TIR [72] (*σ*_3_ is the confining pressure, *σ*_t(d)_ is the variation in axial strength, *β_f_* is the associated orientation of the failure plane, *d* is the weakness plane, *T_w_* is the critical value of normal stress across the weakness plane, and *T_i_* is the tensile strength of intact rock material; the red line represents the failure plane of the specimen).

There are two methods for measuring the tensile strength of rocks: the direct tensile method [66,67] and the indirect tensile method [66,68]. Liao et al. [69] revealed that the direct tensile strength increased with *θ* by conducting direct tensile tests. Nova and Zaninetti [70] and Li and Aubertin [71] established the phenomenological expressions to describe the direct tensile strength of TIR. Lee and Pietruszczak [72] gave a relationship between the tensile strength and failure mode (see Figure 5) of TIR and found that *T_i_/T_w_* = 1 denotes an isotropic sample, and the anisotropy in the tensile strength becomes more pronounced with increasing *T_i_/T_w_*.

**Figure 6:**
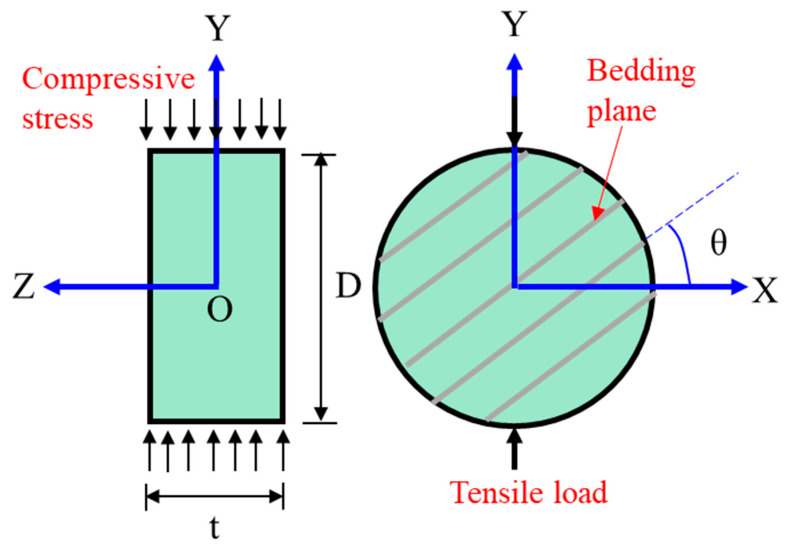
Brazilian test for an anisotropic material [77].

**Figure 7:**
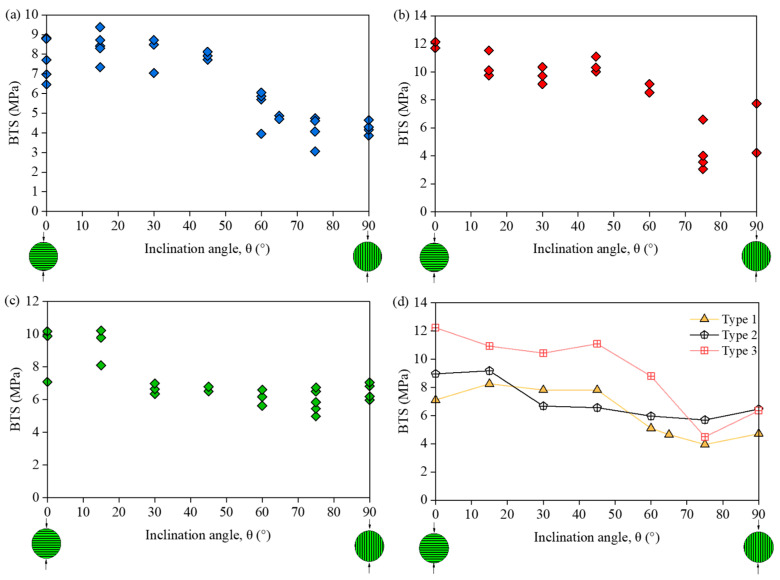
Variations in BTS with *θ* for sandstone types (**a**) 1, (**b**) 2, and (**c**) 3; (**d**) variations in average BTS with *θ* [82].

**Figure 8:**
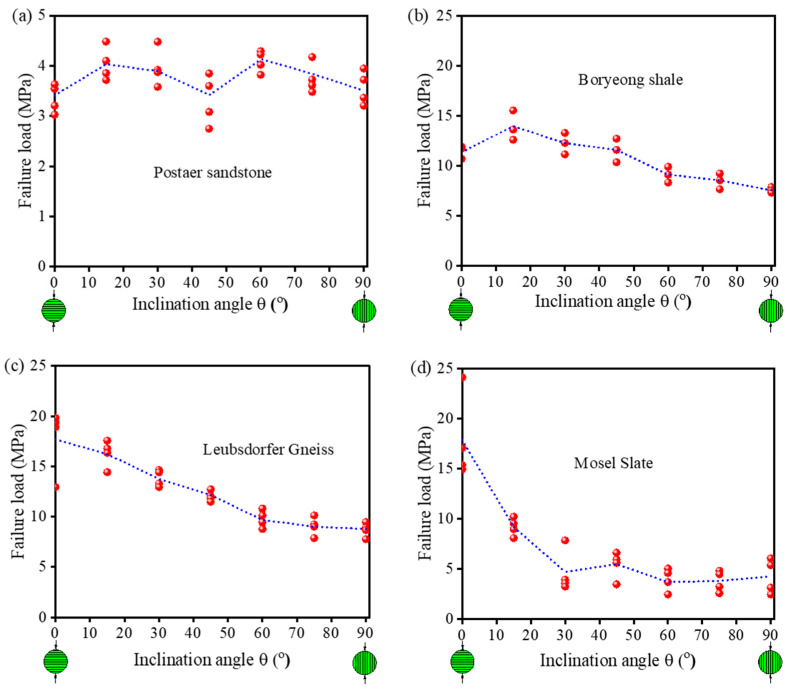
Four variation trends: (**a**) trend 1, (**b**) trend 2, (**c**) trend 3, and (**d**) trend 4 [84].

The Brazilian test, as an indirect tensile test method, is normally used to determine the Brazilian tensile strength (BTS) of TIR. In order to investigate the BTS characteristics of TIR, many scholars have obtained the tensile strength under different bedding angles by conducting the Brazilian test [73,74,75,76], as shown in Figure 6 [77]. Chen et al. [78] found that the BTS of TIR is not a constant but depends on the angle between the bedding plane and the loading direction. Claesson and Bohloli [79] presented a study of an analytical solution of the principal tensile stress (in particular at the center of the rock disc where the crack initiates) for anisotropic (transversely isotropic) rock. Tavallali and Vervoort [77,80,81] obtained the three types of failure modes: the central fracture (*θ* < 45°), transformation from the central fracture to layer activation (45° < *θ* < 60°), and layer activation (*θ* > 60°). As illustrated in Figure 7a–c, Khanlari et al. [82] obtained the variations in BTS as a function of *θ* for 3 sandstone types: type 1 (transition angle 45° < *θ* < 60°, Figure 8a), type 2 (transition angle 60° < *θ* < 75°, Figure 7b), and type 3 (transition angle 15° < *θ* < 30°, Figure 7c). Figure 7d shows that the average BTS at *θ* < 75° is the minimum. Dan et al. [83] found that the degree of anisotropy has a significant effect on the measured BTS. Vervoort et al. [84] summarized four variation trends of a failure load with the bedding angle: little or no variation (trend 1 in Figure 8a), a constant value (0° < *θ* < 45°) followed by a linear decrease (trend 2 in Figure 8b), a linear decrease (trend 3 in Figure 8c), a huge decrease (0° < *θ* < 30°) followed by leveling off (trend 4 in Figure 8d).

### 2.4. Mechanical Properties and Strength Criteria under Triaxial Compression

**Figure 9:**
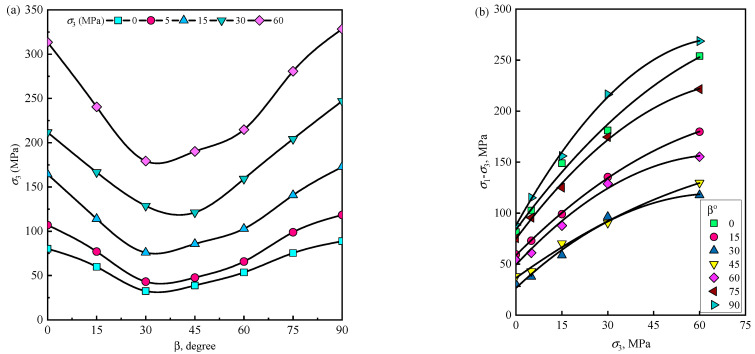
(**a**) Variation in strength with orientation of planes [46], and (**b**) nonlinear strength characteristic of phyllite [46,85].

The deep unexcavated rock mass is in a three-dimensional (3D) stress state. Therefore, the 3D stress characteristics of TIR are of great significance. In the indoor test, the 3D stress characteristics are obtained by conducting the triaxial compression test. The triaxial compression test mainly includes two test methods: the conventional triaxial experiment (*σ*_1_ > *σ*_2_ = *σ*_3_) and the true triaxial experiment (*σ*_1_ > *σ*_2_ ≥ *σ*_3_). The main difference between these two methods is that the three directions of the true triaxial test can be loaded independently. To investigate the triaxial mechanical properties of TIR, Kumar [46] found that the triaxial compressive strength under different confining pressures shows a trend of first decreasing and then increasing with an increasing bedding angle, which is consistent with the changing trend of the UCS with the bedding angle (Figure 9a). Singh et al. [85] noticed that the variation in strength under different bedding angles increases with the confining pressure according to the data of Kumar [46], and the behavior is observed to be nonlinear, as shown in Figure 9b. For transversely isotropic rocks, this conclusion has been confirmed in the literature [86,87,88,89,90,91,92]. According to the statistical results, the triaxial compressive strength of most rock materials has a U-shaped variation trend with the bedding angle, the maximum compressive strength occurs at approximately 0° or 90°, and the minimum compressive strength occurs at approximately 30°.

The strength criteria commonly used in rock mechanics are the Mohr–Coulomb (M-C) and Hoek–Brown (H-B) strength criteria. The M-C strength criterion is a shear failure criterion used to evaluate the strength characteristics of a rock undergoing shear failure. Jaeger [93] proposed a variational cohesion theory based on the M-C criterion. The cohesion in this theory can be considered as a function of the bedding angle, and the internal friction angle is constant. However, Zhao [94] and Si et al. [95] found that the shear parameters of rock alter with a change in the strain rate. They also established the dynamic M-C strength criterion of intact rock. McLamore and Gray [86] regarded the internal friction angle as a function of the bedding angle and modified Jaeger’s variable cohesion criterion. Singh et al. [85] modified the M-C criterion by considering the nonlinear response under different confining pressures. Gu et al. [96] modified the nonlinear M-C criteria for isotropic materials and transversely isotropic unidirectional composites.

The Hoek–Brown strength criterion is an empirical formula obtained from a large number of test results which can reflect the nonlinear trend of change in the rock strength [97]. To accurately evaluate the strength characteristics of TIR, some scholars have proposed various anisotropic failure criteria. Based on the fracture mechanics theory of the isotropic Hoek-Brown strength criterion, Li et al. [98] established the initiation conditions of the bifurcated microcrack along bedding which begins with the meso-fracture mechanism of laminated rocks. Li et al. [99] improved the rock parameters by introducing anisotropy parameters related to the microstructure tensor and loading direction, and they proposed the H–B criterion reflecting the strength anisotropy. Shi et al. [100] developed the H–B criterion to evaluate the triaxial strength characteristic by using the anisotropic index. Through a literature review, it can be found that most of the modified M-C and H-B criteria usually achieve the evaluation of the strength of TIR by modifying the parameters (cohesion, internal friction angle, or rock parameters) of these strength criteria.

## 3. Progress in Experimental Investigation on TIR Rockburst

**Figure 10:**
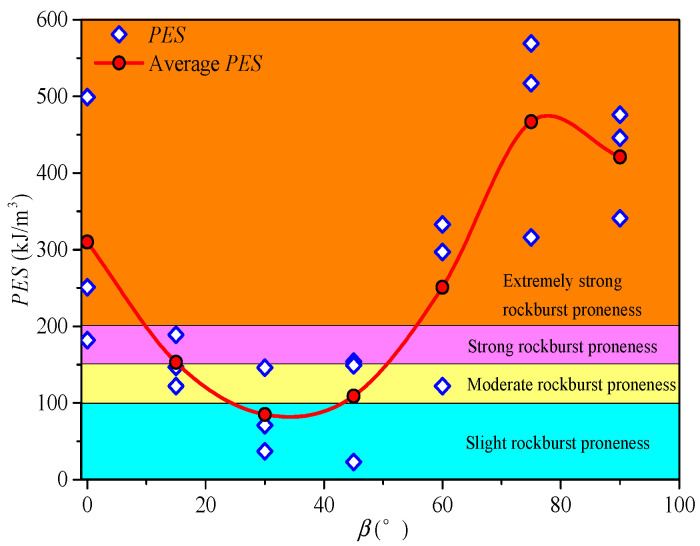
The *PES* and rockburst proneness of phyllite under different bedding angles [1].

In deep underground engineering, the high-stress surrounding rock of deep tunnels or caverns is normally subject to dynamic disturbances or a local stress adjustment, and the stress concentration can be more complex in areas of an abnormal geological structure (e.g., rock mass interfaces and bedding planes) and may induce rockburst. For example, during the excavation of the Neelum–Jhelum (NJ) tunnel in Pakistan, severe and typical rockburst occurred [101,102,103,104]. The rockburst disaster caused the loss of several lives and enormous damage to the construction equipment (e.g., tunnel boring machine) [105]. Therefore, many scholars have conducted extensive studies on rockburst [106,107,108]. For the transversely isotropic rocks, it is important to consider the effects of the cementation strength of bedding planes and the bedding angle on the proneness and severity of rockburst. Si et al. [1] investigated the rockburst proneness of phyllite with different bedding angles under uniaxial compression using the potential energy of the elastic strain (*PES*) index [109,110]. The test results indicate that the average *PES* exhibits a U-shaped variation trend with an increasing bedding angle, as shown in Figure 10. The bedding angle significantly influences the rockburst proneness, and the rockburst proneness of phyllite exhibits the anisotropic characteristics. The same conclusions can also be found from Li and Cui [111]. Yang et al. [112] conducted the single-cyclic loading and unloading uniaxial compression test on phyllite under different bedding angles and obtained that the energy storage limit changes in a “U” shape with the bedding angle.

The underground engineering rock mass is subject to a complex stress path of “three-dimensional high stress + excavation unloading + stress adjustment (dynamic disturbance)” [33,34]. He et al. [113,114] conducted a rockburst simulation experiment on the sandstone with different bedding planes using a modified triaxial apparatus. Their test results show that the bedding angle has an apparent effect on rockburst severity. Under high ground stress, the spatial distribution and structural responses of transversely isotropic rocks should also be considered during rockburst [115,116,117,118]. The research related to deep underground engineering should be consistent with the stress path of the underground rock mass and the environment of the rock mass as much as possible, so that the research results can be well applied to engineering practice. Therefore, rockburst simulation experiments of transversely isotropic rocks under complex stress paths of “three-dimensional high stress + internal unloading + stress adjustment (dynamic disturbance)” should be conducted in the future to reveal the process and mechanism of the rockburst.

## 4. Trend in Experimental Study of TIR

For the transversely isotropic rocks, because there are multiple sets of parallel bedding planes in the rock, the mechanical properties and failure modes of the rocks are affected by the bedding angle, leading to the complexity of the mechanical properties of TIR. In deep underground engineering, the mechanical properties of TIR have an important impact on the stability and failure characteristics of the surrounding rock. Therefore, it is necessary to reveal the mechanical properties and to evaluate the strength under distinct bedding angles. On this basis, for a more effective design of the rock support, it is necessary to study the rock failure characteristics consistent with those under the stress path of real underground engineering as much as possible. The existing problems and development directions in the investigation of transversely isotropic rocks are presented below.

**Figure 11:**
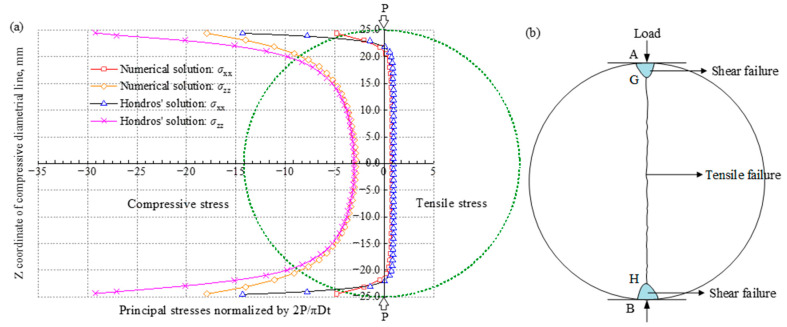
(**a**) Stress distribution [123] and (**b**) transition between shear and tensile failure modes [120] of the specimen in the Brazilian disc split test.

**Figure 12:**
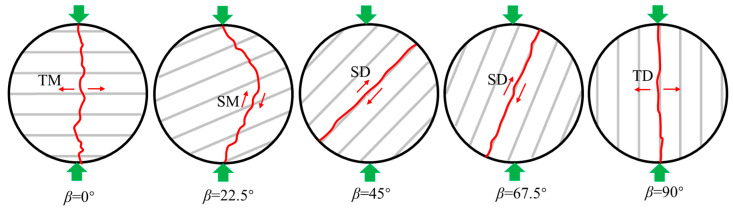
Final failure modes of the specimens with five bedding angles under quasi-static conditions (TM: tensile fracture across discontinuities; SM: sliding across discontinuities; SD: sliding along the bedding; TD: tensile failure along discontinuities) [119].

1. Measuring the Brazilian tensile strength of TIR:

The International Society for Rock Mechanics (ISRM) and the American Society for Testing and Materials (ASTM) suggest using the direct [66,67] and indirect tensile methods [66,68] to measure the tensile strength of rock materials. The stress distribution and failure mode of the specimen in a Brazilian disc split test are shown in Figure 11. It can be seen that compressive stress is distributed at the upper and lower loading edges. After leaving the edges, the *σ*_zz_ along the loading direction still acts as compressive stress, and the *σ*_xx_ normal to loading direction serves as tensile stress and tends to be evenly distributed, as shown in Figure 11a. When the tensile stress reaches the bearing limit, a split failure occurs along the loading direction of the specimen. Theoretically, the failure should initiate from the center of the specimen and develop to both sides, as shown in Figure 11b. However, in the Brazilian disc split test of transversely isotropic rocks, the failure of many specimens does not start from the center and then propagate to the edges on both sides. In addition, the shear crack length ratio in the failure mode is relatively high, as shown in Figure 12 [119]. Therefore, the Brazilian disc test is inappropriate for TIR, as the related results are neither the exact anisotropic solution [120,121,122] nor the actual tensile fracture behavior [69]. It is therefore best to use the direct tensile test method to determine the tensile strength of transversely isotropic rocks.

2. Establishing the strength criteria of TIR:

The M-C strength criterion is a shear failure criterion. In Section 2.4, the modified Mohr–Coulomb criteria for transversely isotropic rocks were introduced. The failure modes of transversely isotropic rocks under distinct bedding angles are very different. For instance, a splitting failure occurred along the bedding plane (*β* = 0°, Figure 4). Therefore, the failure mode under this bedding angle does not satisfy the basic condition of the M-C criterion, and thus the M-C criterion cannot be used to evaluate the rock strength. According to the above analysis, the bedding angle has an important influence on the mechanical properties of TIR. Therefore, the rock damage constitutive model under distinct bedding angles can be established. On this basis, a strength criterion, which is not only suitable for the failure mode transformation but can also comprehensively evaluate the rock strength under different bedding angles, should be established. This will offer important guiding significance for practical engineering.

3. Revealing the influence of mineral particles between bedding planes on rock failure from the microscopic point of view:

For different TIRs, the anisotropy is strong or weak. For some rocks, the bedding plane may have little influence on rock failure, and thus the influence can even be ignored. Why does this phenomenon occur? It is inextricably linked to the mineral particles of the bedding plane and its cementation strength. The bedding plane is generally considered to be a weak surface, i.e., the cementation strength of the bending plane is weaker than the cementation strength between rock particles. Therefore, the strength and failure mode will be affected by a weak bedding surface. In addition, if the cementation strength of the mineral particles between the bedding planes is stronger than that between the rock particles, the existence of the bedding planes does not weaken the rock strength but strengthens the rock strength. Therefore, it is very necessary to investigate the cementation strength of the mineral particles and bedding plane of TIR, and to establish the damage constitutive model of TIR from the microscopic point of view.

4. Investigating the mechanical properties of TIR in complex environments:

In the current research, the mechanical properties of TIR (UCS, tensile strength, and triaxial compressive strength) and the transformation of failure modes have been deeply investigated. In actual engineering, TIR is subject to deep high-stress and complex geological environments, including the underground water-rich layers (acidic, alkaline, or neutral solutions), high ground temperature, high permeability water pressure, etc. Different geological environments will have varying degrees of influence on the failure properties of transversely isotropic rocks. For example, water or dynamic disturbance can weaken the strength of transversely isotropic rocks [124,125,126,127,128]. In addition, due to the different mineral particles between the bedding planes in transversely isotropic rocks, some mineral particles may undergo physical and chemical reactions in acidic or alkaline solutions, thereby affecting the properties of the bedding plane. Through thermal treatment on transversely isotropic rocks (e.g., shale), Masri et al. [129] found that the elastic modulus and compression strength significantly decrease with an increasing temperature. A change in temperature also affects the anisotropic response related to the deformation of bedding planes. The high-temperature thermal treatment has a significant effect on the mechanical properties of TIR [130,131,132,133]. The permeability water pressure has a significant effect on the propagation of cracks in rocks. Zhao et al. [134,135,136,137] investigated the cracking behavior of rock subjected to hydraulic pressure and far-field stress, and they revealed the creep crack mechanism of rock under hydraulic pressure. They also proposed the capacity of a dual-medium model to reveal the damage development in a coal seam under high-pressure water injection. In deep underground engineering, TIR suffers from the coupling effect of high geostress and a complex environment. Therefore, the mechanical properties of TIR under the coupling effect of a stress field and complex environment should be investigated and analyzed. The test results have more guiding significance for practical engineering.

5. Studying the rockburst of TIR under the stress path of “three-dimensional high stress + internal unloading + dynamic disturbance” by true triaxial test:

In deep underground engineering, the unexcavated rock mass is in a three-dimensional stress state. However, excavation changes the stress environment of a rock mass (stress adjustment) [138]. The rock mass after excavation is frequently subject to dynamic disturbance during the construction process, such as blasting, which can lead to rockburst or spalling failure. Therefore, the rock mass is undergoing a complex stress path of “three-dimensional high stress + excavation unloading + stress adjustment (dynamic disturbance)” [33,34]. Si et al. [34] developed a drilling unloading test apparatus and realized a rockburst simulation test following the stress path of “two-dimensional high stress + internal unloading + stress adjustment (dynamic disturbance)”. On this basis, simulation tests on the failure process and characteristics of a tunnel or cavern surrounding rock under complex stress paths should be conducted, and then a constitutive model comprehensively considering the influence of bedding angles and complex stress paths should be established to reveal the failure mechanism of the surrounding rock.

6. Understanding the influence of bedding angle, thickness, and number on the rockburst proneness of TIR:

The rockburst proneness index mainly refers to a kind of indicator to characterize the impact tendency during rock failure based on the mechanical properties of rock. The investigation of rockburst proneness is important for the risk assessment of a rockburst disaster. A variety of discriminating indexes for rockburst proneness are proposed from the perspective of energy, such as the strain energy storage index [139], the energy impact index [140], and the potential energy of the elastic strain index [141]. It has been reported that a certain miscalculation often happens during rockburst proneness discrimination using these indexes [142]. Recently, Gong et al. [143,144,145] first discovered the linear energy storage law, which was proved to be applicable to many materials such as rock, coal [146], and concrete [147] and various test types (the uniaxial compression test [148,149,150,151,152], triaxial compression test [153,154], tension test [144], shear test [155], and three-point flexural loading test [156]). They also established a new rockburst proneness criterion based on the residual elastic energy index, and the accuracy of rockburst discrimination was greatly improved. Based on the linear energy storage law, Gong et al. [157] also proved the rationality of the elastic energy index as a rockburst criterion. It has been pointed out that the mechanical properties of transversely isotropic rocks are affected by the bedding angle, thickness, and number [9]. Therefore, the influence of the bedding angle, thickness, and number on rockburst proneness should be investigated from the perspective of energy. The influence law and mechanism provide an important reference for judging the stability of the surrounding rock and assessing the rockburst risk in practical engineering, and they have certain guiding significance for the support of the surrounding rock.

## 5. Conclusions

This paper summarizes the mechanical properties and rockburst proneness and proposes several prospects for the research of TIR. The conclusions are as follows:

(1) There are many factors that affect the mechanical properties of TIR, which can be divided into two categories: the factors from rock itself and those from the external environment. From the perspective of the rock itself, there are three important factors: the mineral composition, the cementation strength between mineral particles, and the bedding angle. The external environmental factors include the stress, water content, and ground temperature.
(2) The bedding angle and external environment affect the mechanical properties of TIR by changing the cementation strength of the rock. The degree of influence is related to the anisotropy of the bedding plane. A closer cementation strength between the bedding plane and rock particles results in a weaker anisotropy of the rock, and the failure behavior of TIR is less affected by the bedding plane. On the contrary, the obvious weak bedding plane has a significant influence on the mechanical properties and failure mode of the transversely isotropic rock.
(3) The research on deep underground engineering should be consistent with the stress path of an underground rock mass and the environment of the rock mass as much as possible so that the research results can be well applied to engineering practice. Combined with the existing problems in the study of TIR, six research directions considering the coupling effect of complex stress paths and the geological environment are put forward: (1) measuring the Brazilian tensile strength of TIR; (2) establishing the strength criteria for TIR; (3) revealing the influence of mineral particles between bedding planes on rock failure from the microscopic point of view; (4) investigating the mechanical properties of TIR in complex environments; (5) conducting the experimental investigation on the rockburst of TIR under the stress path of “three-dimensional high stress + internal unloading + dynamic disturbance”; and (6) understanding the influence of the bedding angle, thickness, and number on the rockburst proneness of TIR.

## Data Availability

All data that support the findings of this study are included within the article.
